# Complex microsurgical perineal reconstruction after resection of a giant verrucous carcinoma associated with anal fistulas in Crohn’s disease—a unique case report

**DOI:** 10.1007/s00384-020-03569-z

**Published:** 2020-03-17

**Authors:** Denis Ehrl, Markus Rentsch, Nicholas Moellhoff, Nikolaus Wachtel

**Affiliations:** 1 Division of Hand, Plastic and Aesthetic Surgery, University Hospital, LMU Munich, Pettenkoferstr. 8a, 80336 Munich, Germany; 2 Department of General, Visceral and Transplantation Surgery, University Hospital, LMU Munich, Munich, Germany

**Keywords:** Crohn’s disease, Verrucous carcinoma

## Abstract

**Background:**

Crohn’s disease (CD) is a chronic inflammatory disorder which leads to anorectal fistulas. In rare cases, patients develop anal squamous cell carcinoma (ASCC) within these lesions. There is limited literature regarding ASCC in patients with CD. Here, we report on a unique case of advanced verrucous carcinoma (VC), a rare variant of squamous cell carcinoma, developing on the grounds of extensive chronic anorectal fistulas in CD.

**Methods and results:**

A 54-year-old male patient with a 20-year history of CD presented with a large inflammatory tumor at the perineal region with multiple discharging perianal fistulas. Histopathological analysis of the perineal mass revealed a VC. Subsequent surgery with radical tumor resection and terminal colostomy resulted in a large perineal cavity and a partially exposed sacrum. The defect extended to a total of 35 × 25 × 25 cm. Reconstruction was achieved through a two-step approach. A first surgical step established an arteriovenous (AV) loop in the upper thigh. Subsequently, a free latissimus dorsi (LD) myocutaneous flap was harvested and anastomosed with the AV loop, allowing for satisfactory closure of the defect and reconstruction of the perianal and perineal region.

**Conclusion:**

Radical surgical excision with negative margins is the therapy of choice for VC. This case report demonstrates a curative treatment option with special emphasis on the reconstructive possibilities of a unique case of extended perianal and perineal VC associated with chronic anorectal fistulas in CD.

## Introduction

Crohn’s disease (CD) is a chronic inflammatory disorder that may affect the whole gastrointestinal mucosa. Anorectal fistulas are a common manifestation in approximately 23% to 38% of cases of CD and in 60% of patients with CD of the anus or rectum [1–3]. In rare cases, patients develop anal squamous cell carcinoma (ASCC) within these lesions. Exemplary, Ky et al. reported an incidence of about 0.4% in their 14-year follow-up study of over 1000 patients with CD [4].

There is limited literature regarding ASCC in patients with CD; most reports consist of small series or single cases [2, 4–6]. Treatment options include radiation, chemotherapy, and surgical resection [2, 5]. Here, we report on a unique case of advanced verrucous carcinoma (VC), a rare, low-grade, and well-differentiated variant of squamous cell carcinoma (SCC), developing on the grounds of extensive chronic anorectal fistulas in CD. The aim of our report was to contribute to the limited literature regarding reconstructive and curative treatment options in this small patient group.

## Methods and results

**Fig. 1:**
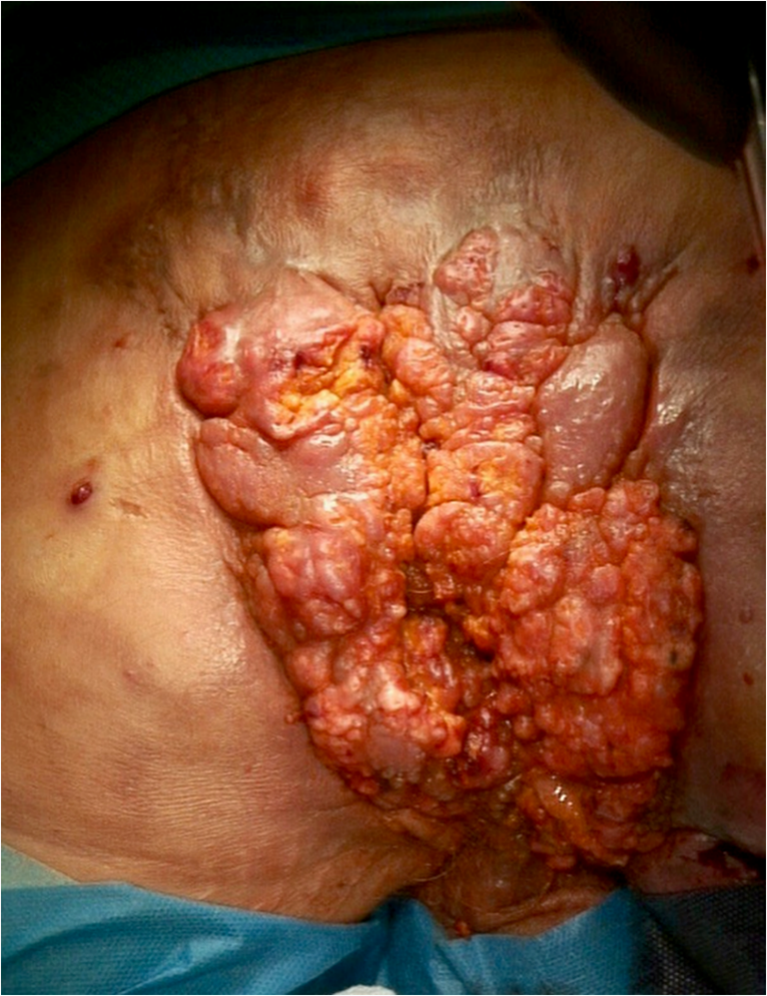
Extensive inflammatory perineal tumor with multiple perianal fistulas (20 × 10 cm)

**Fig. 2:**
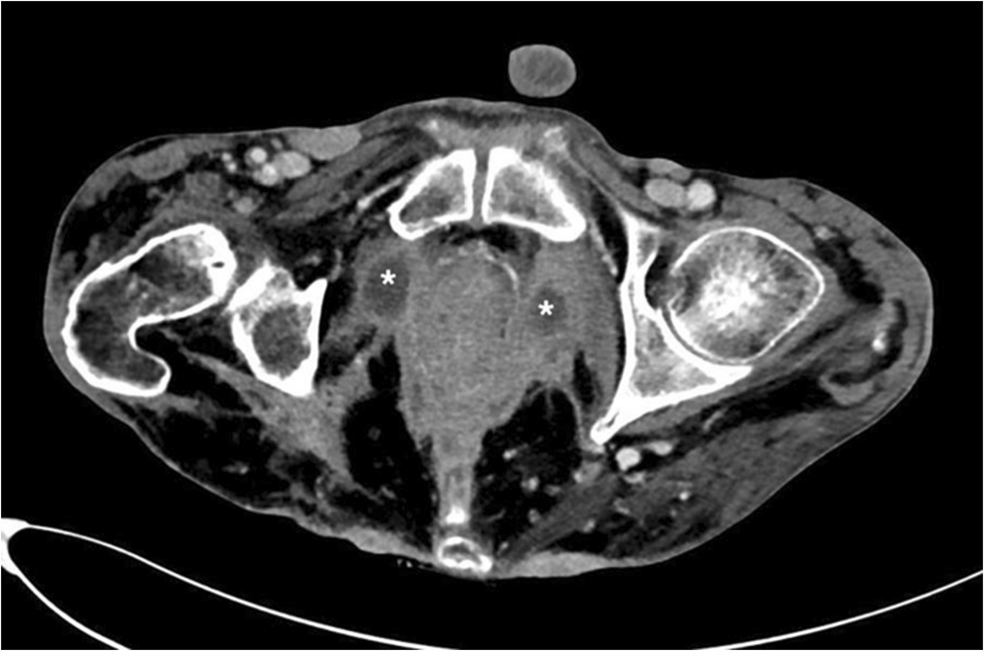
CT scan of the perineal and perianal region showing multiple abscesses (marked by an asterisk)

Due to discharge of stool through perianal fistulas and massive weight loss of 40 kg over a period of 9 months, a multi-morbid 54-year-old male patient with a 20-year history of CD was admitted to the emergency room. Past surgical history included multiple drainage incisions of perineal abscesses, as well as a transversostomy due to stenosis of the descending colon. Moreover, the patient reported to be a heavy smoker (40 PY) and suffered from alcoholic cardiomyopathy (left ventricular ejection fraction (LVEF) of approximately 20%). Physical examination revealed severe cachexia (BMI < 17.0 kg/m^2^) and a large inflammatory tumor at the perineal region with multiple discharging perianal fistulas (Fig. 1). A computed tomography (CT) scan showed multiple perineal and perianal abscesses (Fig. 2). CT-guided, percutaneous drainages of the abscesses were inserted; the patient was started on antibiotic treatment and hypercaloric enteral as well as parenteral diet. Histopathological analysis of the perineal mass revealed a VC, with negative in situ hybridization for human papillomavirus (HPV).

**Fig. 3:**
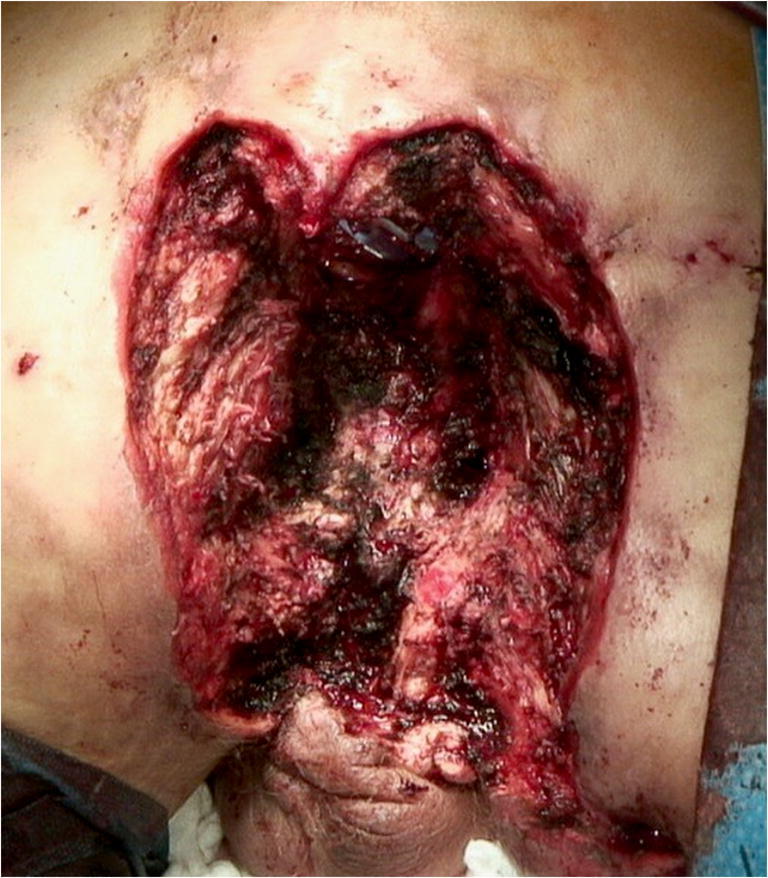
Abdominoperineal resection and excision of the perineal tumor resulted in a wound with a cavity of approximately 35 × 25 × 25 cm

Subsequently, abdominoperineal resection, wide excision of the perineal tumor, terminal colostomy, and occlusion of the pelvic floor by omentoplasty and VICRYL® mesh was performed. Surgery resulted in a large wound with a cavity of approximately 35 × 25 × 25 cm and a partially exposed sacrum (Fig. 3). The surgical site was temporarily covered and conditioned with vacuum-assisted closure (VAC) therapy.

**Fig. 4:**
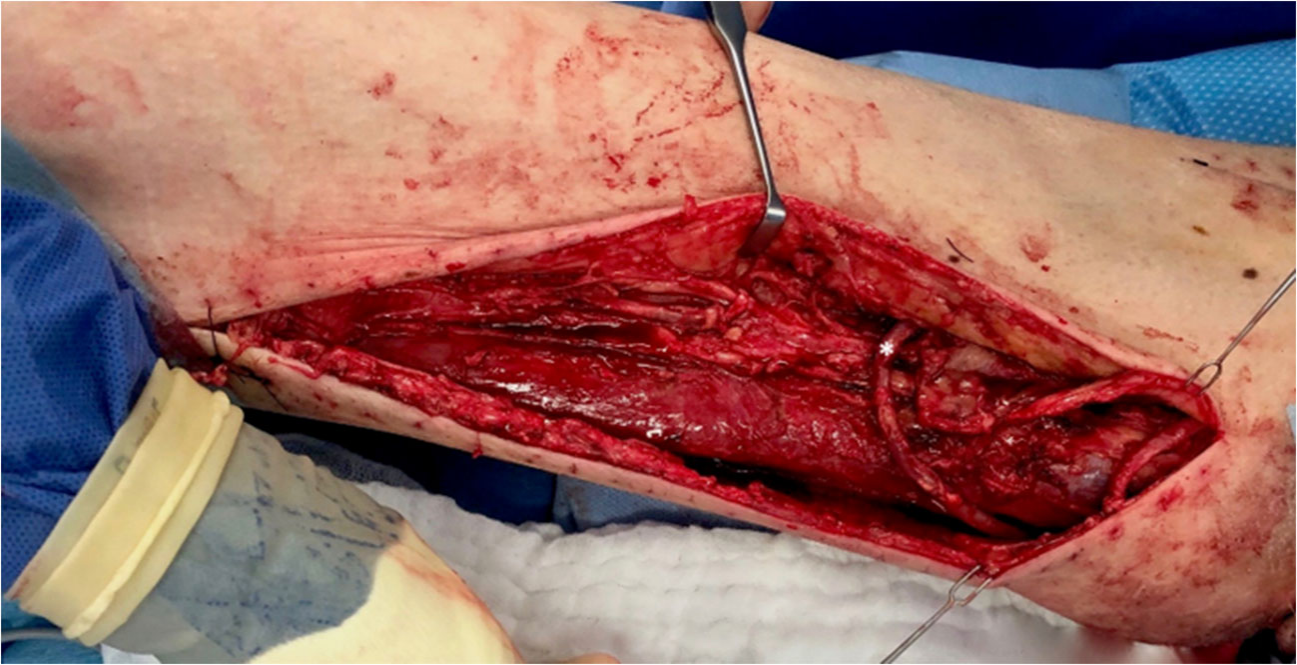
Fistula (AV loop) between the great saphenous vein and the lateral circumflex femoral artery (marked by an asterisk)

**Fig. 5:**
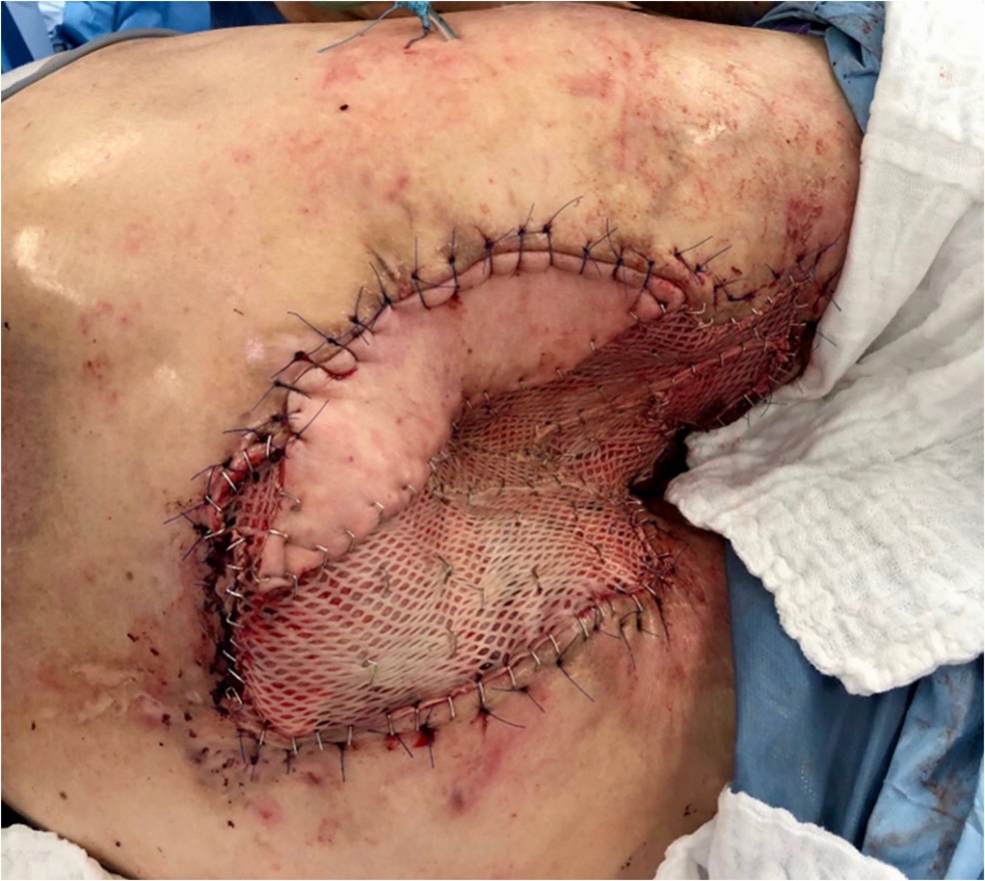
Intraoperative view with free latissimus dorsi myocutaneous flap inserted to reconstruct the perineal defect, the muscular part of the flap was covered with split-thickness skin grafts

Three subsequent surgical debridements were necessary to prepare the surgical site for the final wound closure. Prior to defect reconstruction, due to poor quality of local perforator vessels and bilateral resection of superior and inferior gluteal vessels during abdominoperineal resection, a first surgical step established an arteriovenous (AV) loop. For this, the right great saphenous vein was mobilized and anastomosed to the lateral circumflex femoral artery (Fig. 4). Subsequently, the patient returned to the operating room and the AV loop was transferred to the perineal region. A free latissimus dorsi (LD) myocutaneous flap was harvested (size, 38 × 28 cm). The apex of the AV loop was cut, and arterial and venous anastomoses were completed in standard fashion with interrupted sutures. The muscular part of the flap was covered with split-thickness skin grafts from the lateral thigh. Two 12 Charrière, negative-pressure surgical drainages were inserted between the flap and the underlying tissue (Fig. 5). Additionally, the complete split-thickness skin grafted muscular part of the flap was covered with large polyurethane foam and consistent negative pressure therapy for 5 days was started. After removal of the negative pressure system, we observed a partial peripheral necrosis of the flap (9 × 3 cm). Therefore, 11 days after defect coverage, the patient returned to the operating room; excision of necrosis and primary wound closure with a locoregional rotational flap was performed. Subsequent wound healing was without complications, sutures were removed 21 days post-surgery.

**Fig. 6:**
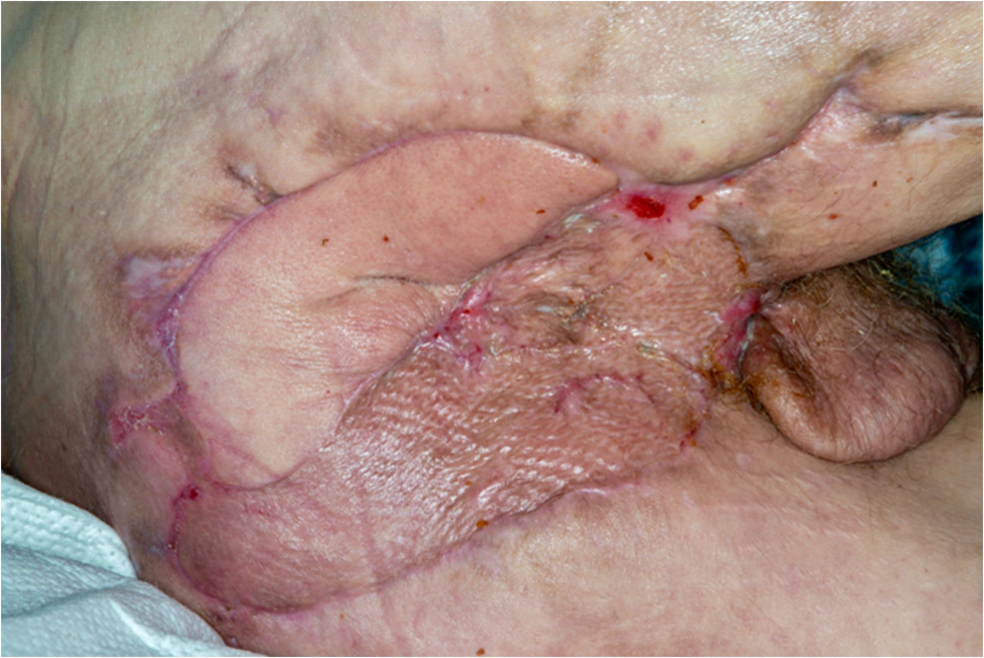
Result 7 weeks postoperatively

After reconstructive surgery, the patient was transferred to the Intensive Care Unit (ICU) and consequently placed in a lateral decubitus position. Mobilization was commenced 7 days after free flap transfer. During the course of the subsequent treatment, the patient developed acute cardiac decompensation (LVEF of < 10%) and consequently acute liver failure, which significantly prolonged the inpatient stay. After stabilization and treatment of cardiac failure, LVEF and liver function gradually recovered. The patient was equipped with a LifeVest® and discharged 69 days after his last surgical procedure (Fig. 6).

## Conclusion

ASCCs are a rare complication of perianal fistulas due to CD. In their report, Benjelloun et al. review all published cases since 1983 (*n* = 21) [2]. Additionally, a case series including seven patients was recently reported by Lightner et al. [5]. VCs are rare, exophytic, low-grade, well-differentiated SCCs characterized by their locally invasive growth and low incidence of metastasis [7–9]. Infection with HPV and exposure to chronic inflammation have been implicated as etiologic factors [7, 8, 10, 11]. Following extensive research of the literature, the presented patient is the first published case describing VC associated with chronic anorectal fistulas in CD.

Radical surgical excision with negative margins is the therapy of choice for VC [7, 12, 13]. Due to severe inflammation with multiple abscesses of the perianal and perineal region, abdominoperineal resection with wide tumor excision was the only curative treatment option for the patient (Figs. 1 and 2). Surgery resulted in a large cavity with a partially exposed sacrum (Fig. 3). A common and well-established technique for reconstruction of perineal defects is the vertical rectus abdominis muscle (VRAM) flap [1, 14–16]. However, in this case, extensive scarring after previous laparotomy, in addition to the size of the defect, prevented reconstruction using a VRAM flap. Moreover, other locoregional flaps (e.g., bilateral gracilis muscle flaps) were not sufficient in size to cover the defect. Similarly to other authors who published reconstructive procedures with equally large perineal or pelvic defects, we decided to use an ipsilateral free LD myocutaneous flap for reconstruction of the pelvic floor [17, 18]. To ensure postoperative adherence of the flap, two negative-pressure surgical drainages were inserted between the flap and the underlying tissue. Additionally, negative pressure therapy was performed for several days, exerting continuous and controlled pressure towards the pelvic floor.

Previous external resection of superior and inferior gluteal vessels as well as the poor quality of local perforator vessels required the installment of an AV loop that could later be mobilized into the defect—thus allowing adequate perfusion for the planned flap [19–21]. Due to the localization of the defect and the mandatory positioning of the patient after LD transfer, we decided to create a fistula between the great saphenous vein and the lateral circumflex femoral artery (Fig. 4).

Reconstructive surgery involving free flap tissue transfer in combination with an AV loop has been reported as single- or two-stage procedure [19, 20, 22]. In the current case, cardiomyopathy, in particular, prevented extensive duration of anesthesia. We therefore planned a two-stage approach to divide the perineal reconstruction into two more manageable procedures which significantly reduced time of continuous anesthesia. Nevertheless, the patient suffered from cardiac decompensation after free LD flap transfer, which significantly prolonged hospitalization. A single-stage procedure with extensive anesthesia therefore seems no viable option for multi-morbid patients requiring complex surgical reconstruction.

A free LD myocutaneous flap provides an adequate solution for the reconstruction of large perineal and perianal defects. Moreover, the installment of an AV loop should be considered if locoregional vessels are of inadequate caliber. In the current case, this procedure enabled the curative therapy in a patient with CD, suffering from extensive anorectal fistulas in combination with anal VC.

## Data Availability

Not applicable.
