# Correction to: Complex microsurgical perineal reconstruction after resection of a giant verrucous carcinoma associated with anal fistulas in Crohn’s disease—a unique case report

**DOI:** 10.1007/s00384-021-04042-1

**Published:** 2021-10-30

**Authors:** Denis Ehrl, Markus Rentsch, Nicholas Moellhoff, Nikolaus Wachtel

**Affiliations:** 1 Division of Hand, Plastic and Aesthetic Surgery, University Hospital, LMU Munich, Pettenkoferstr. 8a, 80336 Munich, Germany; 2 Department of General, Visceral and Transplantation Surgery, University Hospital, LMU Munich, Munich, Germany

## Correction to: International Journal of Colorectal Disease (2020) 35:1337–1341 https://doi.org/10.1007/s00384-020–03,569-z

The article “Complex microsurgical perineal reconstruction after resection of a giant verrucous carcinoma associated with anal fistulas in Crohn’s disease—a unique case report”, written by Denis Ehrl, Markus Rentsch, Nicholas Moellhoff and Nikolaus Wachtel, was originally published Online First without Open Access. After publication in volume 35, issue 7, page 1337–1341 the author decided to opt for Open Choice and to make the article an Open Access publication. Therefore, the copyright of the article has been changed to © The Author(s) 2021 and the article is forthwith distributed under the terms of the Creative Commons Attribution 4.0 International License, which permits use, sharing, adaptation, distribution and reproduction in any medium or format, as long as you give appropriate credit to the original author(s) and the source, provide a link to the Creative Commons licence, and indicate if changes were made. The images or other third party material in this article are included in the article’s Creative Commons licence, unless indicated otherwise in a credit line to the material. If material is not included in the article’s Creative Commons licence and your intended use is not permitted by statutory regulation or exceeds the permitted use, you will need to obtain permission directly from the copyright holder. To view a copy of this licence, visit http://creativecommons.org/licenses/by/4.0.

